# Plant-produced recombinant cytokines IL-37b and IL-38 modulate inflammatory response from stimulated human PBMCs

**DOI:** 10.1038/s41598-022-23828-z

**Published:** 2022-11-14

**Authors:** Igor Kolotilin

**Affiliations:** Solar Grants Biotechnology Inc, London, ON Canada

**Keywords:** Immunology, Molecular biology, Plant sciences, Biologics, Expression systems, Biotechnology, Plant biotechnology, Molecular engineering in plants

## Abstract

Affordable therapeutics are vitally needed for humans worldwide. Plant-based production of recombinant proteins can potentially enhance, back-up, or even substitute for the manufacturing capacity of the conventional, fermenter-based technologies. We plastome-engineered a tobacco cultivar to express high levels of two “plantakines” — recombinant human cytokines, interleukins IL-37b and IL-38, and confirmed their native conformation and folding. Assessment of their biological functionality was performed ex vivo by analyzing the effects exerted by the plantakines on levels of 11 cytokines secreted from human peripheral blood mononuclear cells (PBMCs) challenged with an inflammatory agent. Application of the plant-produced IL-37b and IL-38 in PBMCs stimulated with Lipopolysaccharide or Phytohaemagglutinin resulted in significant, and in particular cases—dose-dependent modulation of pro-inflammatory cytokines secretion, showing attenuation in two-thirds of significant level modulations observed. Plantakine treatments that increased inflammatory responses were associated with the higher dosage. Our results demonstrate feasibility of manufacturing functional recombinant human proteins using scalable, cost-effective and eco-friendly plant-based bioreactors.

## Introduction

Plants make a lot of sense as production platforms for all kinds of biologics. Photosynthetic capacity allowing autotrophic growth renders plants the most energy-efficient and cost-effective platform for manufacturing of various recombinant proteins, secondary metabolites and other assorted small molecules, as plants require only three abundantly available raw input ingredients for biosynthesis—carbon dioxide, water and sunlight. Hence, the initial part of the manufacturing process, the "upstream production" that generates the biomass accumulating the desired product ensues significant costs savings, eliminating the need for construction, maintenance and operation of fermenter facilities^1–3^. Benefits to “downstream production” steps of the process, where the desired product is extracted and purified are also recognized for plant-based systems, with some of the bottlenecks being addressed in recent studies^4–6^. Additional advantages of exploiting plants as single-use, clean and biodegradable bioreactors for production of recombinant proteins include inherent safety due to inability of mammalian pathogens to propagate in plant tissue and virtually unlimited scalability of plant-based production^7,8^.

Since the emergence of the first reports of successful genetic transformation of plants and the expression of recombinant heterologous proteins of human origin in transgenic plants, tremendous technological advances were achieved in the “molecular pharming” field, with the first FDA-approved pharmaceutical for human use in 2012, taliglucerase alfa, produced in carrot cells^9^. Today several biopharmaceuticals on the market are sourced from plants and a few biotechnology companies around the world use plant-based production platforms in their manufacturing processes^3,6,10^. Plant-based bioreactors could facilitate making more affordable many biologic drugs in use today and provide a source of therapeutics supplied locally, which can be very beneficial in the context of developing nations, or when global supply chains are disrupted^6,11^.

Among the methodologies used for plant-based recombinant protein manufacturing, plastome-engineered plants possess several advantageous features as a platform, simply generating extraction-ready biomass from seed. Plastome-engineered plants can express and accumulate very high yields of the desirable product and, thus, can represent the most cost-effective production route^12–15^. We set to demonstrate the feasibility of plastome-engineered plant bioreactor platform for production of biologically active recombinant human cytokines. Based on our preliminary screens searching for valuable proteins with a potential for prolific expression in plastids, we engineered the plastome of a low-alkaloid tobacco cultivar to produce “bioreactor lines” expressing mature forms of two “plantakines”—human interleukins IL-37 (isoform b, IL-37b) and IL-38, both characterized as anti-inflammatory cytokines^16,17^. IL-37b and IL-38 belong to the IL-1 family of 11 interleukins, 7 of which are pro-inflammatory^18^. Both IL-37b and IL-38 function in regulation/mitigation of human inflammatory responses; a plethora of studies demonstrated central involvement for IL-37b and IL-38 in immunity and disease and, therefore, as potential candidates for development as therapeutic agents^19,20^. The created plastome-engineered bioreactor lines produced up to ~ 1 g of the recombinant protein per 1 kg of fresh leaf biomass. After confirmation of their correct folding via protein-specific ELISAs, we assessed the biological activity of the plant-produced IL-37b and IL-38 in ex vivo experiments by monitoring the response to inflammatory agents (IAs) in freshly isolated cultured human peripheral blood mononuclear cells (PBMCs), manifested in the levels of secreted inflammatory cytokines.

PBMCs are the central and crucial components of the immune system that brings forth a response to intruder pathogens, as well as identifies and fights own body cells that have undergone malignant transformation (cancer). PBMCs are an assorted mixture of highly specialized immune cells, PBMCs population is comprised of a multitude of immune cell types including lymphocytes (~ 85%), monocytes (~ 15%) and dendritic cells (< 1%)^21^. In vitro and ex vivo human PBMCs studies are ubiquitous in cell biology and immunology research and an important biotechnological tool in developing new therapeutics and diagnostics^22–26^. We hypothesized that by monitoring the inflammatory response in IA-stimulated PBMCs we could study the effects of the plantakines (and their active concentrations) exerted on the levels of specific inflammatory markers.

We report significant modulation of inflammation responses from PBMCs stimulated with different IAs as a result of treatments with the plant-produced IL-37b and IL-38. We observed attenuation of levels of several secreted inflammatory cytokines, generally consistent with the previous reports characterizing the biological activity of IL-37b and IL-38 as anti-inflammatory. Both plantakines exerted dose-dependent modulations of PBMCs responses, leading at different concentrations to either inhibition or enhancement of secretion of some of the inflammatory markers monitored. In addition, different IAs brought about different magnitude of inflammatory responses reflected in levels of cytokines secreted from the stimulated PBMCs, confirming a similar experimental outcome reported recently. Thus, our study validates applicability of the plant-based production platform for cost-efficient and eco-friendly manufacturing of functional recombinant human cytokines in large quantities.

## Results

### Monomers, dimers and multimers of IL-37b and IL-38 accumulate in engineered chloroplasts

**Figure 1:**
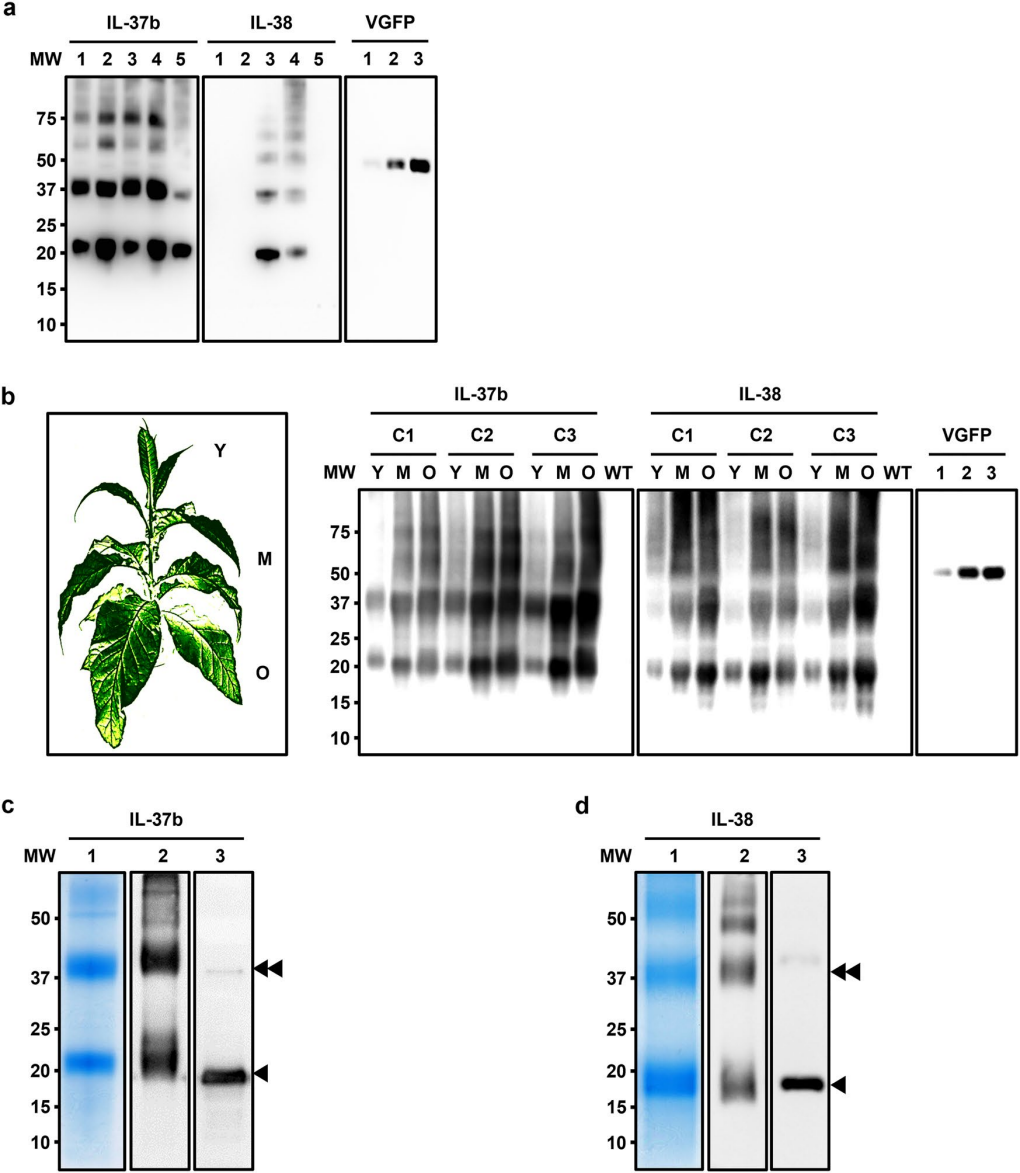
Expression and Purification of the Plantakines IL-37b and IL-38. (**a**) SDS-PAGE and Western blots of samples from crude leaf tissue extracts of the primary transplastomic clones generated for expression of plantakines IL-37b and IL-38 tagged with a HIS-tag at the C-terminus. Numbers 1–5 for each IL-37b and IL-38 represent extracts (~ 100 µg fresh leaf tissue) from different clones; clones 1, 2 and 5 for IL-38 show no expression. VGFP (EGEH^27^) is a HIS-tagged GFP variant used as quantifiable control protein; lanes 1, 2 and 3 represent 12.5, 25 and 50 ng, respectively. All blots probed with the same anti-His tag antibody. (**b**) Left panel: Schematic representation of a greenhouse-grown bioreactor plant assessed to determine the spatial expression patterns of the cytokines by sampling young (Y), mature (M) and old (O) leaves; Right panel: Three clones (C1, C2, C3) for each bioreactor line expressing either IL-37b or IL-38 were sampled (~ 1 mg fresh leaf tissue in lane) and assessed with Western blots. Wild-type (WT) tobacco extracts were used as negative controls. VGFP was used as quantifiable control protein; lanes 1, 2 and 3 represent 12.5, 25 and 50 ng, respectively. (**c**) Lanes 1 and 2 both contain ~ 1 µg of the purified plantakine IL-37b, SDS-PAGE and stained (lane 1) or Western-blotted and probed with anti-IL-37 antibody (lane 2) along with 500 ng of bacteria-produced human recombinant IL-37b as a control (lane 3). (**d**) Lanes 1 and 2 both contain ~ 1 µg of the purified plantakine IL-38, SDS-PAGE and stained (lane 1) or Western-blotted and probed with anti-IL-38 antibody (lane 2) along with 500 ng of bacteria-produced human recombinant IL-38 as a control (lane 3). Molecular weight marker (MW) ladder is in kiloDaltons. Single black triangle arrows depict the monomers of the plantakines of the predicted molecular sizes (20.3 kDa and 18.3 kDa for IL-37b and IL-38, respectively), double arrows depict the dimers. Higher molecular weight multimeric structures are also detectable. Original blots/gels used for Figure 1 compilation are presented in Supplementary Material.

We engineered the plastome transformation constructs to produce IL-37b and IL-38 as mature peptides (V46–D218 for IL-37b, C2–W152 for IL-38), optimizing the expression by selecting suitable *cis*-acting regulatory genetic elements and using plastid-preferable codons (data not shown). Screening for prolific producer lines of IL-37b and IL-38 identified the best configurations of plastid expression cassettes by examining their crude leaf tissue extracts with Western blots (Fig. 1a). Two bioreactor lines were selected and grown in greenhouse to maturity, expressing the recombinant human IL-37b and IL-38 at ~ 1 g and 0.75 g, respectively, per 1 kg of fresh leaf tissue. Interestingly, prevalent amounts of both plantakines were found to accumulate in older leaves, demonstrating significant stability of these recombinant proteins in the chloroplasts (Fig. 1b). We observed large amounts of the monomeric forms, as well as the dimerized and multimerized forms of the cytokines IL-37b and IL-38 in the crude leaf extracts and in samples after purification; the dimers (and higher molecular weight multimers) were very stable and detectable in SDS-PAGE analyses gels even after harsh denaturing conditions of the sample preparation. That observation was in stark contrast to the bacteria-produced recombinant IL-37b and IL-38 counterparts available commercially, that predominantly presented the monomeric forms of the cytokines when used as controls in Western blot experiments using specific antibodies (Fig. 1c,d). Placement of the HIS-tag at the N-terminal had no effect on the formation of dimers/multimers for both expressed cytokines (data not shown).

### Plantakines bioactivity assessment—experimental design

The bioactivity of our plantakines IL-37b and IL-38 was assessed by monitoring the secretion of 11 cytokines, generally regarded as inflammatory markers, from PBMCs stimulated with an IA. Freshly isolated human PBMCs were subjected to various treatments—combinations of the IAs with the plantakines at different concentrations. Two different model IAs were used, either the bacterial lipopolysaccharides (LPS) or a lectin from *Phaseolus vulgaris* (phytohaemagglutinin, PHA); each of the IAs was applied onto cells separately, each IA was applied at two concentrations: LPS at 150 and 300 pg/mL; PHA at 5 and 10 µg/mL. Each IA at each concentration was applied in combination with one of the two plantakines, each of them at three different concentrations: 1, 10 and 100 ng/mL of the monomeric forms present in the purified extracts. Also included were treatments comprised of either IA at their lower concentrations, in combination with both plantakines at 10 ng/mL concentration in order to assess possible synergistic effects. Cells with only IAs applied represented the reference (positive controls for each concentration), cells without any treatment represented the basal level (negative control). The levels of eleven different pro-inflammatory cytokines secreted into the medium from the PBMCs—GM-SCF, IFNγ, TNFα, IL-1α, IL-1β, IL-6, IL-8, IL-22, IL12, IL-17 and IL-10 were compared between the treatments and the controls. We applied generalized estimating equation (GEE) model for the statistical data analysis. This statistical approach allows for nested observations and was used to test the effects of the plantakines IL-37b and IL-38 and their dosage in the context of IA-stimulated PBMCs inflammatory responses.

### Different IAs bring about different magnitude of inflammatory responses from PBMCs

**Figure 2:**
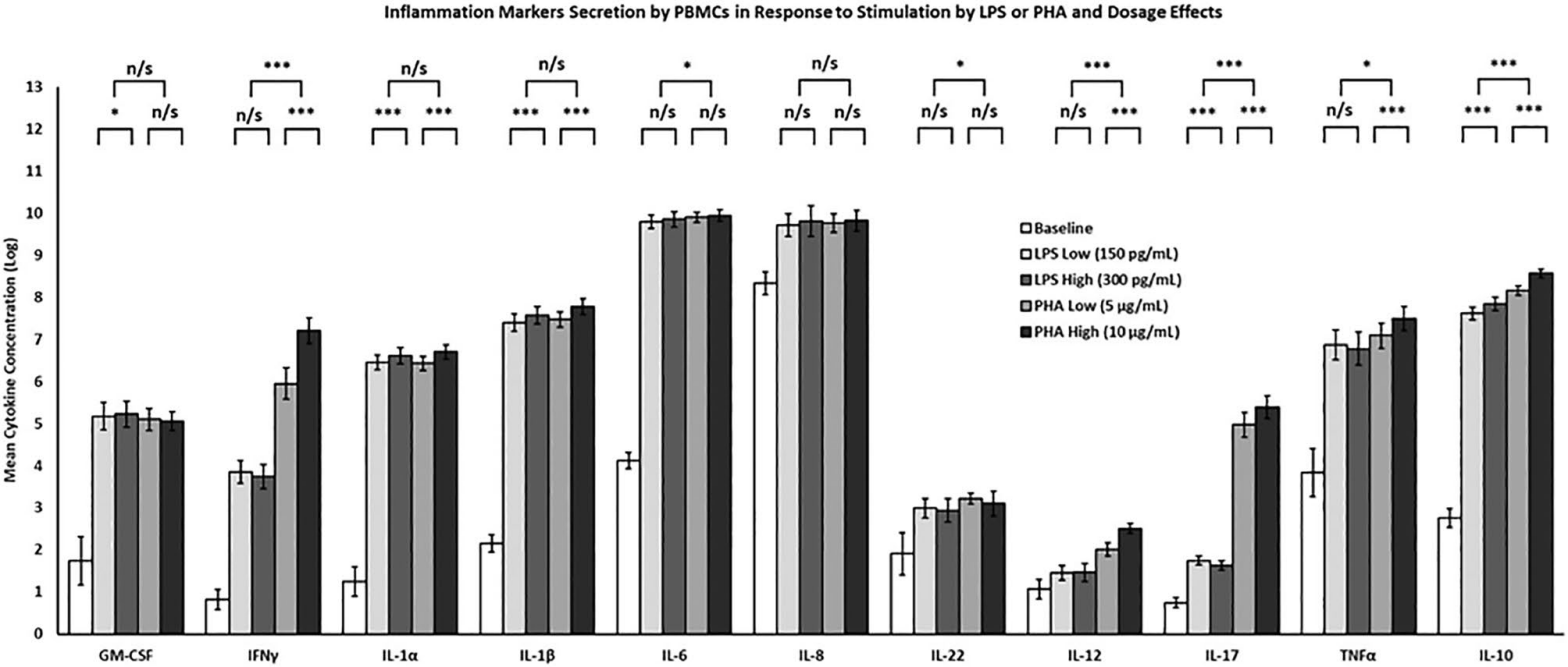
Differences in levels of inflammatory cytokines secreted from PBMCs in response to stimulation with either LPS or PHA and effects of IA dosage. Statistically significant differences are indicated for mean concentration values (+/− SEM) obtained for each marker under LPS or PHA stimulation, as well as differences between the low and high doses within the applied IA. Baseline secretion from PBMCs for each of the markers was significantly different (*p* < 0.001) than the mean concentration secreted in response to either IA. Legend: n/s—no significant difference; *—significant difference, *p* < 0.05; ***—significant difference, *p* < 0.001.

In order to validate the obtained data, as well as to gain insights into the quantitative and qualitative differences in the PBMCs’ inflammatory responses between the two IAs tested, we first compared the mean values for each monitored secreted cytokine elicited by either LPS or PHA. The magnitude of the general responses from the stimulated PBMCs, manifested in levels of the secreted pro-inflammatory cytokines was found significantly different between the two IAs. PHA elicited stronger response in 7 out of 11 pro-inflammatory cytokines monitored; namely, IL-17, IFNγ, TNFα, IL-12, IL-22, IL-10 and IL-6 displayed, respectively, 2930%, 1240%, 55.7%, 118%, 21.5%, 86.4% and 10.1% higher levels, compared with the LPS-elicited levels (*p* < 0.001—*p* < 0.05, Fig. 2). The levels of GM-CSF, IL-8, IL-1α and IL-1β showed no statistically significant difference between the IAs in our experiments. Further dissection of the data focusing on the differences in levels of the inflammatory markers between the lower and higher doses of each IA revealed that the higher dose of PHA elicited higher levels of IFNγ (+ 249%), IL-1α (+ 30%), IL-1β (+ 35%), IL-12 (+ 65%), IL-17 (+ 54%), TNFα (+ 49%) and IL-10 (+ 51%), compared with the lower dose. Higher dose of LPS brought about higher secreted levels of GM-CSF (+ 5%), IL-1α (+ 17%), IL-1β (+ 20%) and IL-10 (+ 26%), while secretion of IL-17 decreased (− 12%).

### Plant-produced IL-37b and IL-38 modulate inflammatory responses from IA-stimulated PBMCs

To gain insight into the bioactivity of the plantakines IL-37b and IL-38 exerted on IA-stimulated PBMCs we compiled the data generated from treatments with observed modulatory effects on secreted inflammatory markers. GEE analysis was performed four times, for each combination of the IA and its concentration, for each secreted cytokine monitored, generating statistically significant (*p* < 0.05) level modulations displayed in 118 treatments out of the total 286 treatment combinations assessed. Plantakines exerted statistically significant modulatory effects on the levels of secreted inflammatory cytokines in 67 and 51 treatments that occurred in LPS- and PHA-stimulated PBMCs, respectively (Table 1). Collectively, treatments with plantakines IL-37b and IL-38 resulted in more profound anti-inflammatory activity in LPS-stimulated PBMCs rather than PBMCs stimulated with PHA, as only 10 treatments resulted in increased secretion of inflammatory cytokines in LPS-stimulated PBMCs, while decreased secretion was observed in 57 treatments. In contrast, secretion of inflammatory markers in PHA-stimulated PBMCs was suppressed in 17 treatments with plantakines and increased in 34. Notably, all the treatments with the simultaneous application of both IL-37b and IL-38 brought about increases in secretion of inflammatory cytokines under stimulations with either IA, while separate applications of the plant-produced IL-37b or IL-38 suppressed secretion of inflammatory cytokines in 44 and 30 treatments, and increased it in 12 and 22, respectively. Fewer treatments with plantakines caused suppression of inflammatory cytokines secretion and the numbers of treatments where inflammatory cytokines secretion increased grew in association with a higher concentration of either IA used to stimulate the PBMCs (Table 1).

**Table 1:** Modulation in levels of inflammatory cytokines secreted from IA-stimulated PBMCs with applied IL-37b and IL-38 plantakines treatments.

PBMCs stimulation IA								
LPS				PHA				
150 pg/mL		300 pg/mL		5 µg/mL		10 µg/mL		IA concentration
IL-37b	IL-38	IL-37b	IL-38	IL-37b	IL-38	IL-37b	IL-38	Plantakine treatment
286								All treatments combinations
118								Treatments with statistically significant modulation (*p* < 0.05)
67				51				
39		28		27		24		
10				34				Increased secretion
5*		5		17**		17		
1	1	3	2	3	7	5	12	
57				17				Decreased secretion
34		23		10		7		
18	16	11	12	9	1	6	1	

*—includes 3 treatments combinations with simultaneous application of both plantakines.

**—includes 7 treatments combinations with simultaneous application of both plantakines.

**Figure 3:**
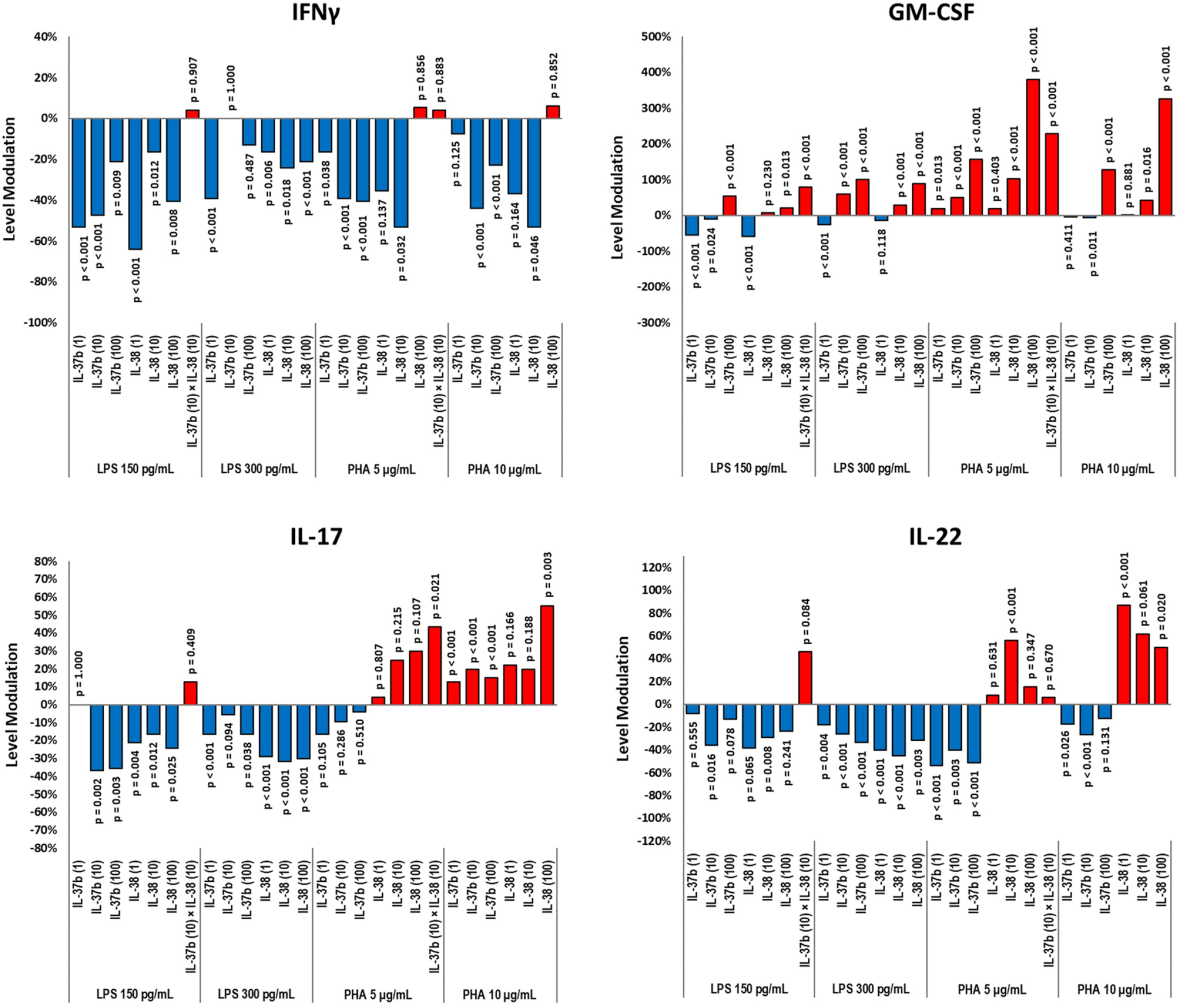
Modulations of secretion of select inflammatory markers (IFNγ, GM-CSF, IL-17 and IL-22) by stimulated PBMCs after treatments with different doses of the plant-produced recombinant IL-37b and IL-38. The effects of the treatments were calculated as percentages (Level Modulations, the “Y” axis) of secretion modulation with its probability value, derived from comparison with the positive control levels (0%, the “X” axis) at the corresponding IAs concentrations for each inflammatory marker monitored.

We further analysed the changes in levels of the secreted inflammatory cytokines from the stimulated PBMCs resulting from treatments with different doses of the plantakines IL-37b and IL-38. For each inflammatory marker monitored, the outcomes of the treatments were calculated as percentages of secretion modulation with its probability value in comparison with the positive controls at the corresponding IAs concentrations (Fig. 3; Table SI, Figure S1 in Supplementary Materials). The modulatory effects of the plantakines IL-37b and IL-38 could be observed on the levels of most of the monitored secreted inflammatory cytokines elicited with either LPS or PHA, showing attenuation in 63% of the statistically significant modulations. IL-37b attenuated levels of IFNγ, IL-1α, IL-1β, IL-22, IL-17 and TNFα in LPS-stimulated PBMCs, the effect could be seen at all the concentrations examined, levels of IFNγ and IL-22 were also reduced by IL-37b in PHA-stimulated PBMCs (Fig. 3; Table SI). Increases in secretion of the inflammatory markers were mostly observed after treatments with higher concentrations of plantakines, whereas 66% of the treatments where secretion increases occurred were associated with the highest dose of plantakines, followed by 28% associated with the intermediate dose and 6% linked to the lowest dose. Unexpectedly, IL-37b at all 3 concentrations brought about an increase in IL-17 secreted from PBMCs stimulated with PHA at 10 µg/mL, similar increases were observed for IL-1α and GM-CSF levels with IL-37b at 100 ng/mL. Modulation of GM-CSF levels by both IL-37b and IL-38 displayed dose-dependent character: at low concentrations (1 ng/mL) both plantakines attenuated GM-CSF levels by more than 50% in PBMCs stimulated with 150 pg/mL LPS, while 100 ng/mL plantakines concentration increased the levels of GM-CSF, those increases observed more profoundly at LPS 300 pg/mL concentration (Fig. 3). Both plantakines boosted GM-CSF in PHA-stimulated PBMCs: at higher concentrations (100 ng/mL) IL-37b brought about 155.9% and 127.8% increases in GM-CSF levels at 5 µg/mL and 10 µg/mL PHA stimulation, respectively, and IL-38 showed 380.5% and 326.6%, *p* < 0.001. Interesting, a combination of both plantakines, each at concentration 10 ng/mL exerted a 228.6% (*p* < 0.001) increase in secreted GM-CSF levels, pointing out a possible synergistic effect from the simultaneous application, since when applied separately on PBMCs with the same 5 µg/mL PHA stimulation, plantakines IL-37b and IL-38 modulated GM-CSF levels to increase 50.5% and 103.3%, respectively (*p* < 0.001). Simultaneous applications of both plantakines resulted in increased secretion of several pro-inflammatory cytokines from PHA-stimulated PBMCs, IL-1α, IL-1β, IL-12, IL-17, TNFα and IL-10 displayed, respectively, 35.0%, 35.0%, 134.5%, 43.3%, 61.6%, and 43.3% increased levels (*p* < 0.001—*p* < 0.05). Notably, only insignificant modulation of IL-6 and IL-8 levels was observed, yet, when plantakines IL-37b and IL-38 were applied at the lowest concentration (1 ng/mL), statistically significant attenuation (− 9.5% for IL-6 elicited at 150 pg/mL LPS, *p* = 0.012, and − 28.5% for IL-8 elicited at 300 pg/mL LPS, *p* = 0.032) was detected, aligned with the anti-inflammatory functions expected from IL-37b and IL-38 (Table SI).

## Discussion

In the present study we engineered the tobacco plant plastome to produce green bioreactors capable of manufacturing profuse amounts of two functional recombinant human cytokines, IL-37b and IL-38, mainly known as anti-inflammatory modulators of immune responses^16,28^. To our best knowledge, this is the first report describing such a prolific expression and production of both recombinant human cytokines in their active forms in plants, a previous successful attempt to produce IL-37b in tobacco via nuclear genome transformation reported much lower yields, while there are no reports on IL-38 production in plants hitherto^29^. Very interesting is the fact that the penultimate amino acid (the second amino acid in the peptide chain after the initiating Methionine) of the IL-38 peptide is Cysteine, which was underlined, along with Histidine, as the strongest instability-conferring penultimate amino acid for protein expression and accumulation in plastids, leading the researchers to propose existence of an N-terminus-dependent protein degradation pathway in plastids^30^. Our bioreactor lines produced IL-38 peptide with the penultimate Cysteine at 8–10% of the total soluble protein in the leaf tissue, contradicting the proposed model. Further, the same transformation construct expressing an IL-38 peptide variant with an added penultimate Serine, which was reported as a stabilizing penultimate amino acid, reached similar levels of IL-38 accumulation (data not shown), suggesting that the proposed N-terminus-dependent protein degradation pathway in chloroplasts is either limited in its processing capabilities, or involves additional unknown regulatory factors that specifically direct degradation of select proteins^30^.

The accumulated dimerized (and multimerized) forms of the plantakines IL-37b and IL-38 in the crude leaf extracts and in purified samples constituted ~ 50% of the entire recombinant protein yields, contrasting the results of successful recombinant production studies of IL-37b and IL-38 proteins in bacteria, which never reported dimerization of the purified cytokines in their SDS-PAGE analyses, even when the purified IL-37b was concentrated by ultrafiltration^31–33^. This observation suggests that the chloroplast stroma compartment, accumulating the synthesized recombinant proteins, provides a beneficial milieu of internal conditions/chaperones/scaffolds assisting the folding of these cytokines and promoting their further dimerization and multimerization. It is also reasonable to assume the remarkable stability of those dimer/multimeric forms of the cytokines in plastids, since the highest levels of accumulation were observed in older leaves. Bacteria-produced IL-37b was shown to form dimers at nanomolar concentrations and tetramers at higher concentrations, which greatly diminished its bioactivity, suggesting a mechanism of activity regulation through monomer/dimer equilibrium and leading to an engineered monomeric IL-37b variants with much stronger biological activity^34,35^. Those monomeric variants, however, along with the natural mature recombinant IL-37b peptide showed appearance of minor bands that corresponded to the dimer size in SDS-PAGE analyses and further investigation of these protein structures is needed^34^. Also, intriguing is the question whether the formation of dimers/multimers contributes to the overall stability of those cytokines against proteolysis, thus enabling the proposed mechanism of self-regulation and in situ preservation in a stable inactive form in the intercellular space, where the local IL-37b concentration reached the dimerization constant values^34^. A mechanism of bioactivity regulation through the dimer formation, similar to that of IL-37b, was proposed for IL-38 in a recent review^28^; however, no scientific reports are available addressing this subject. Further structural studies will answer the question whether IL-38 can be engineered into a stable, bioactive monomer, similarly to IL-37b.

Bodily inflammatory processes are a part of innate immune responses, promoted by pro-inflammatory cytokines released from the cells of the immune system as a reaction to the presence of an inflammatory agent or stimuli. Secreted levels of 11 well-characterized inflammatory cytokines, namely GM-SCF, IFNγ, TNFα, IL-1α, IL-1β, IL-6, IL-8, IL-22, IL12, IL-17 and IL-10 were monitored in our experiments with freshly isolated PBMCs subjected to treatments with two different IAs in combination with two plant-produced anti-inflammatory cytokines IL-37b and IL-38 at different concentrations. This experimental setup allowed for focusing on the biological effects exerted by the plantakines IL-37b and IL-38 via a direct comparison of the levels of pro-inflammatory cytokines secreted from IAs-stimulated PBMCs with or without the plantakines treatments at corresponding concentrations (Tables 1, SI).

Although PHA and LPS bind to completely different sets of receptors, both IAs seem to trigger varying signal transduction cascades, particularly leading to the activation of NF-κB, which plays an essential role in regulating the expression of genes linked to innate immunity and inflammatory responses^36,37^. Seven out of 11 inflammatory cytokines monitored in our study displayed significantly higher secretion from PBMCs stimulated with PHA rather than LPS, IL-17 secretion varied more than 30-fold (Fig. 1). Remarkably, our findings strongly corroborate a recent report stating that after PHA-stimulation human PBMCs secreted significantly higher levels of inflammatory cytokines, compared to stimulation with LPS, which apparently failed to notably induce IL-17, TNFα and IL-12^38^.

The modulatory effects exerted on levels of the inflammatory markers secreted from IA-stimulated PBMCs confirmed the biological activity of plant-produced IL-37b and IL-38. Statistically significant modulations occurred in both LPS- and PHA-stimulated PBMCs due to treatments with the plantakines (Table 1). For quenching inflammation, treatments with plantakines appeared to be more effective in LPS-stimulated PBMCs, where 85% of all treatments resulted in attenuations of the levels of secreted inflammatory markers, while only 30% of treatments attenuated inflammatory markers in PHA-stimulated PBMCs, implying varying efficacy and specificity of the anti-inflammatory action under different stimuli. Attenuated, rather than increased secretion of inflammatory cytokines occurred 3.5 and 1.7 times more frequently in treatments with IL-37b and IL-38, respectively, in accord with the proposed role for IL-37b as a primary and fundamental inhibitor of inflammation^16,39^. Treatments combining both plantakines, however, resulted in increased secretion of inflammatory markers under either IA; also, with higher concentrations of either IA applied for PBMCs stimulation, treatments with plantakines suppressive of secretion of inflammatory cytokines became scarcer, while more treatments caused increased inflammatory secretion (Table 1). These observed phenomena are inexplicable at this point and require further scientific exploration.

Levels of the inflammatory markers secreted from the stimulated PBMCs displayed distinct and varying patterns of modulation following application of treatments with the plant-produced IL-37b and IL-38 (Fig. 3; Table SI). The number of treatments that triggered an increased, rather than attenuated secretion, was higher only in 2 among the 11 inflammatory cytokines monitored, namely GM-CSF and IL-12, indicating general anti-inflammatory effects exerted by the treatments with plantakines in IA-stimulated PBMCs. Notable were the differences in the magnitude and the scope of the modulation, reflected in the outcomes of the treatments being either an attenuation or an increase of the inflammatory secretion levels: an attenuation of the secretion was the outcome of 74 treatments, averaging − 28% level reduction, while increases in secreted levels of inflammatory cytokines, observed in 44 treatments, displayed 79% on average. Among the 11 cytokines monitored, only the levels of IFNγ exhibited consistent attenuation from treatments with either plantakine in PBMCs stimulated with either IA, displaying also the strongest attenuation observed in our experiments (− 63.9%, *p* < 0.001), exerted by application of 1 ng/mL IL-38 in PBMCs stimulated with 150 pg/mL LPS. In contrast, GM-CSF levels were 3 times more frequently increased, rather than attenuated by treatments with the plantakines, with the strongest increase reaching 380.5%, *p* < 0.001, upon application of 100 ng/mL IL-38 in PBMCs stimulated with 5 µg/mL PHA (Table SI). Strikingly, the plant-produced IL-37b and IL-38 both exerted dose-dependent regulation of GM-CSF secreted levels, bringing about attenuation at low concentrations, while causing increases at high concentrations. Although both IL-37b and IL-38 are generally characterized as anti-inflammatory cytokines active in quenching inflammation^17,40^, studies have reported that recombinant unprocessed IL-38 could increase inflammatory cytokine IL-6 production in human macrophages in response to LPS or IL-1β stimuli^41,42^. In addition, IL-37b was reported to increase TNFα production in higher concentrations and *Candida*-induced IL-17 production was reportedly blocked by low concentrations of IL-38, while higher doses of IL-38 induced more IL-17 production, a pattern which resembled IL-37b bioactivity^41,43^.

Both IFNγ and GM-CSF are crucial cytokines for activation/differentiation of myeloid cell populations^44,45^ and their nuanced regulation by the plant-produced IL-37b and IL-38 may serve as a primer for future studies to discern novel patterns in PBMCs inflammatory responses. Interesting also to note that statistically significant attenuation of IL-6 and IL-8, two profound inflammation markers monitored in our study^46,47^ was only detected upon applications of low concentrations of the plantakines, aligned with the anti-inflammatory functions expected from IL-37b and IL-38.

In conclusion, we developed plastome-engineered, low-alkaloid tobacco bioreactor lines for cost-efficient and prolific production of two functional human cytokines with profound anti-inflammatory properties, IL-37b and IL-38, which are underlined as prospective therapeutic agents. Our explorative study demonstrated that the plantakines exerted significant modulation of levels of secreted cytokines involved in inflammatory responses monitored in IA-stimulated PBMCs, indicating a dose-dependent mode of action and general attenuation of several secreted inflammation markers. Enhancement of several pro-inflammatory cytokines, associated with higher concentrations of the plantakines applied in treatments was also observed, revealing novel patterns of inflammation regulation by IL-37b and IL-38. Different magnitude of responses from PBMCs were seen in levels of secreted cytokines elicited by different IAs, where PHA elicited stronger response than LPS in levels of most secreted cytokines monitored. Cumulatively, our results demonstrate feasibility of producing functional human recombinant cytokines in plants and further promote the accelerated adoption of plant-based manufacturing of various recombinant proteins by biotechnology industries.

## Methods

### Plastome engineering

Plastome-engineered bioreactor lines expressing recombinant human IL-37b and IL-38 (UniProt identifiers Q9NZH6 and Q8WWZ1, respectively) in their mature forms (V46–D218 for IL-37b, C2–W152 for IL-38), each bearing a C-terminal hepta-HIS-tag, were produced by Igor Kolotilin for Solar Grants Biotechnology Inc.

All permissions for including the plant material/samples were obtained from Solar Grants Biotechnology Inc. All experimental research work in this study, including the collection of plant material, comply with relevant institutional, national, and international guidelines and legislation.

### Recombinant protein extraction and purification

Total soluble proteins from fresh leaf tissue of the bioreactor lines were extracted as described^48^. In short, flash-frozen leaf tissue was milled into powder with pestle and mortar and then 5 volumes of the extraction buffer (1XPBS, pH = 7.4) was added, complemented with 10 µg/mL leupeptin 2 mM PMSF and 2% PVPP. After filtration through Whatman #1 paper, the extract was centrifuged twice for 15 min at 13,000× *g* in 4 °C. The recombinant proteins were purified from the cleared extract utilizing the C-terminal His-tag and the immobilized metal ion-affinity chromatography (Cytiva Life Sciences™ His SpinTrap™, Cat. No. 28932171), dialysed against 1XPBS, pH = 7.4 and filtered through 0.22 µm (EMD Millipore, Cat. No. SLGV004SL) to obtain sterile solutions.

### SDS-PAGE and Western Blots

SDS-PAGE and Western Blots experiments were performed as described previously^48^. Briefly, the clarified extract was mixed in a proper proportion with 5XSample Buffer (50 mM Tris, pH 8.0, 1% SDS, 20 mM DTT, glycerol) and the mixture was boiled at 99 °C for 30 min, after which was cooled down to room temperature and loaded into the gel wells. Anti-His-tag antibodies (GenScript, Cat. No. A00186-100), as well as the monoclonal antibodies against human IL-37b and IL-38 (MyBioSource Inc., Cat. No. MBS 7600509 and R&D Systems, Cat. No. DY9110-05, respectively) were used to detect the blotted proteins according to manufacturers’ recommendations; bacteria-produced recombinant human IL-37b (R&D Systems, Cat. No. 7585-IL-025) and IL-38 MyBioSource, Cat. No. MBS635478) were used as the positive controls. Western blots membranes were visualized using the enhanced chemiluminescence (ECL) detection kit (GE Healthcare, Cat. No. RPN2232) and imaged with the DNR Bio-Imaging System MicroChemi (RANCOM A/S, Birkerød, Denmark). Densitometry was performed using the TotalLab TL 100 software (Nonlinear Dynamics, Durham, NC).

### ELISA experiments

For confirmation of the native folding and quantitation analyses, protein-specific ELISA experiments with the plantakines IL-37b (Invitrogen™, Cat. No. LS885210322) and IL-38 (R&D Systems Inc., Cat. No. DY9110-05) were performed according to manufacturers’ recommendations. BioTek Instruments (VT, USA) Epoch Microplate Spectrophotometer was used to acquire numerical ELISA data.

### Experiments with PBMCs—general design and multiplex cytokine analysis

Sample collection, experimental protocols and all methods were performed in accordance with relevant guidelines and regulations. After isolation, the PBMCs from each donor separately were counted, plated in equal numbers per well and stimulated for 24 h with the applied treatments. Control wells on the plate contained media only (as basal level controls), and LPS and PHA (as positive controls at their corresponding concentrations). After the 24 h treatments stimulation the PBMCs’ culture supernatants were used in the multiplex Luminex platform-assisted analysis of the concentrations of the 11 secreted pro-inflammatory cytokines.

The whole blood from 5 random human donors was collected in ACD Vacutainer tubes and immediately processed for isolation of PBMCs by gradient density centrifugation using Lympholyte. The freshly isolated PBMCs from each donor separately were cultured in a volume of 200 µL at a concentration of 1.25 × 10^6^ cells/mL in 96-well plates (~ 250,000 cells/well) in an incubator set at 37 °C, 5% CO2 and > 80% humidity. PBMCs were treated with one of two IAs at 2 concentrations each (LPS at 150 and 300 pg/mL; PHA at 5 and 10 µg/mL) in combination with two test items (plant-produced IL-37b or IL-38) at three concentrations each (1, 10 and 100 ng/mL), based on the monomeric form amounts estimated with densitometry (ImageJ). Each treatment was tested in triplicates. Control wells contained media only (negative control, basal level of detection) and the examined IAs at both tested concentrations as the positive controls. The PBMCs were pre-incubated with the test items^41^, stimulated with the IAs and incubated for 24 h after the stimulation for analysis of the levels of the secreted cytokines that were determined in the culture supernatant using a multiplex immunoassay (MAGPix^®^, Luminex), analytes were selected from the Milliplex panel HCYTOMAG-60K, sensitivity range of 3.2–10,000 pg/mL. All parameters of the Milliplex panel cytokines analysis using Luminex platform were validated (Millipore-Sigma).

### Statistical analysis

Non-linear logarithm transformation was performed to address non-normality of distribution of Luminex-derived numeric data. Generalized estimating equation (GEE) method was used in the analysis of nested (correlated) structure of the data. Secreted inflammatory cytokine values were used as dependent variables and test item (plantakines) with dosage as independent predictors (factors). Separate analyses were conducted for each of the 11 monitored cytokines and each inflammatory agent/concentration combination. Analysis was performed using SPSS software version 27 using the level of significance 0.05 (*p* values < 0.05 are reported as statistically significant).

## Supplementary Information

Supplementary Information 1.

Supplementary Information 2.

Supplementary Information 3.

Supplementary Information 4.

## Data Availability

All data generated and analyzed during this study are included in this published article and its Supplementary Information files. Enquiries regarding availability of the generated plant bioreactor lines (seeds) should be sent to igor.k@sgbiotec.com.

## Abbreviations

GM-CSF: Granulocyte macrophage-colony stimulating factor
IL: Interleukin
PBMCs: Peripheral blood mononuclear cells
LPS: Lipopolysaccharide
PHA: Phytohaemagglutinin
