# Supplementary material for: Plant-produced recombinant cytokines IL-37b and IL-38 modulate inflammatory response from stimulated human PBMCs
Source: Sci Rep. 2022 Nov 14;12:19450. doi: 10.1038/s41598-022-23828-z (PMC9663505; doi:10.1038/s41598-022-23828-z)
Supplement: Supplementary file 2 — Supplementary Information 2. [file 41598_2022_23828_MOESM2_ESM.pdf]

Supplementary Material – Figure S1 with legend.

Manuscript: Plant-produced Recombinant Cytokines IL-37b and IL-38 Modulate Inflammatory Response from Stimulated Human PBMCs.


Igor Kolotilin,
Solar Grants Biotechnology Inc., London, Ontario, Canada.
igor.k@sgbiotec.com


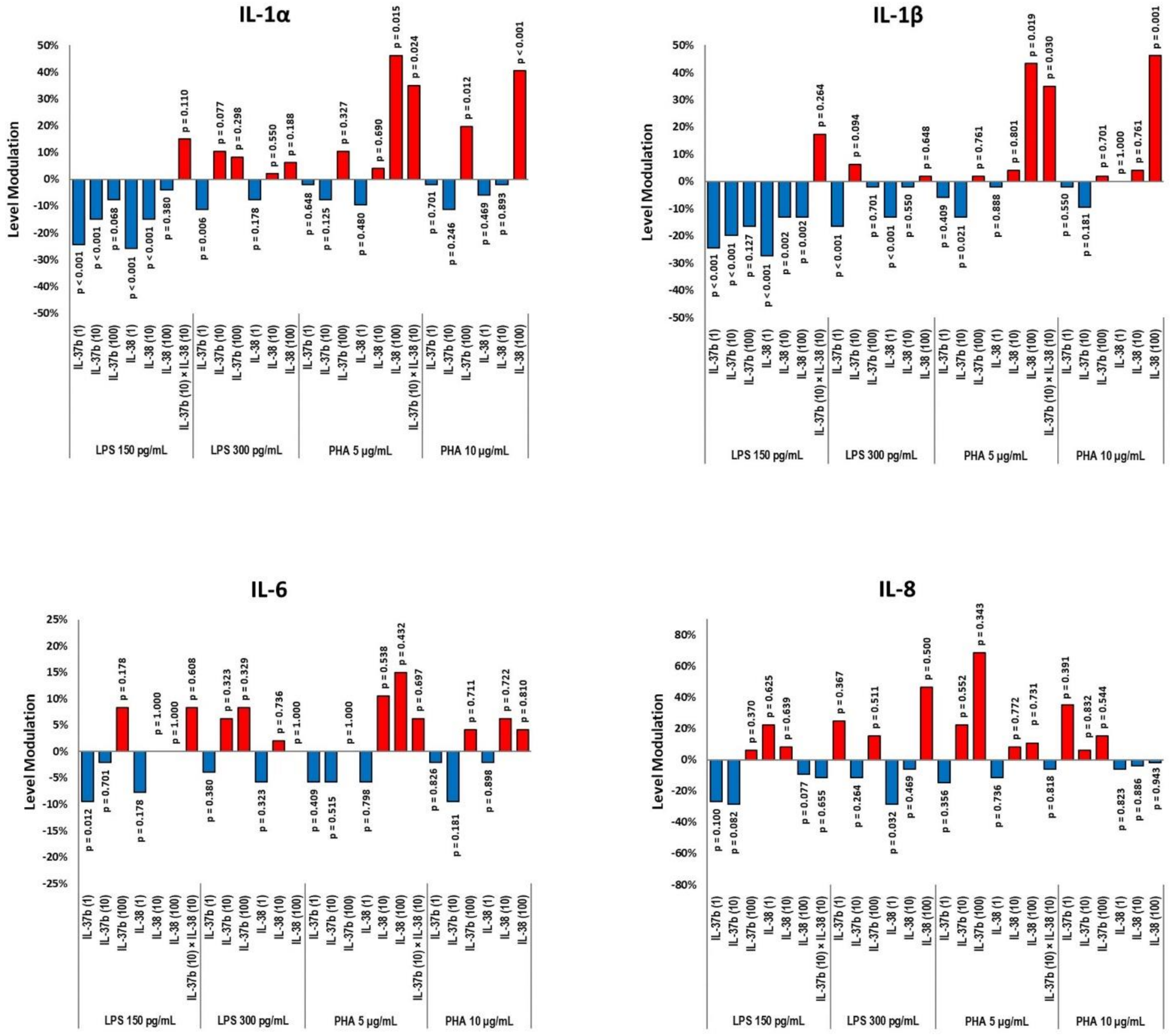

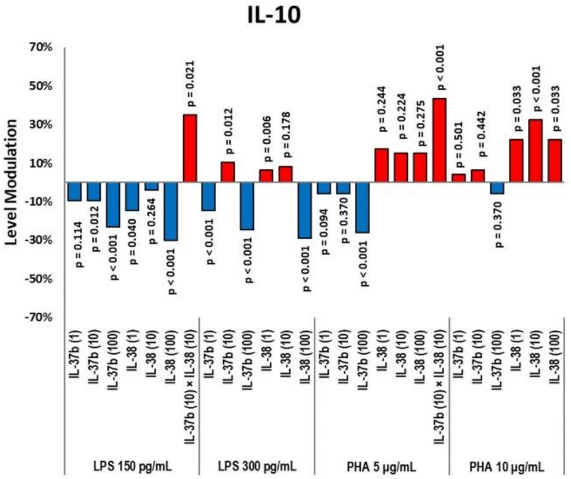


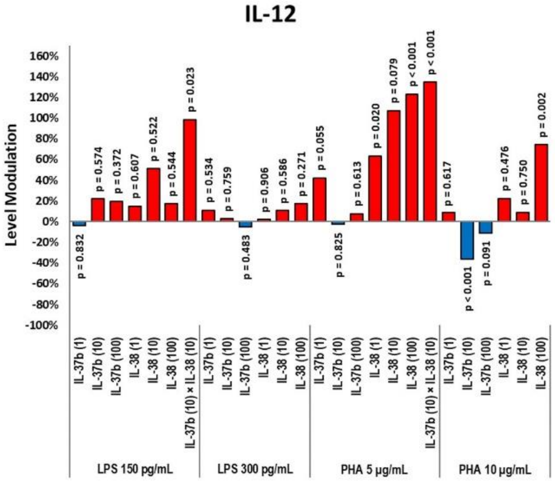


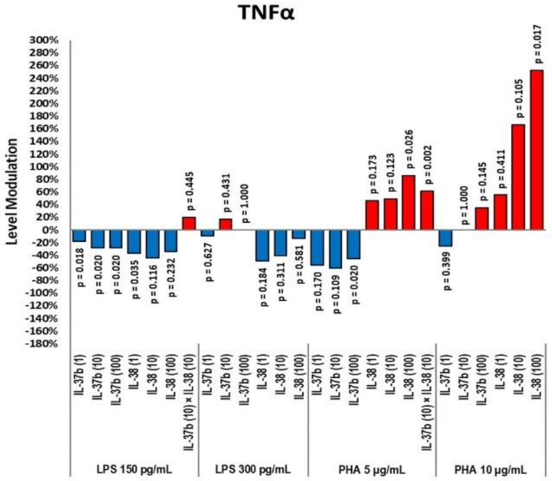


Figure S1. Modulations of inflammatory markers secretion by stimulated PBMCs after treatments with different doses of the plant-produced recombinant IL-37b and IL-38. The effects of the treatments were calculated as percentages (Level Modulations, the “Y” axis) of secretion modulation with its probability value in comparison with the positive controls (0%, the ”X” axis) at the corresponding IAs concentrations for each inflammatory marker monitored.
