# Supplementary material for: Plant-produced recombinant cytokines IL-37b and IL-38 modulate inflammatory response from stimulated human PBMCs
Source: Sci Rep. 2022 Nov 14;12:19450. doi: 10.1038/s41598-022-23828-z (PMC9663505; doi:10.1038/s41598-022-23828-z)

Supplementary Material – Original images and figure compilation process.

Manuscript: Plant-produced Recombinant Cytokines IL-37b and IL-38 Modulate Inflammatory Response from Stimulated Human PBMCs.

Igor Kolotilin,
Solar Grants Biotechnology Inc., London, Ontario, Canada.
igor.k@sgbiotec.com

Figure 1.

Complete image compilation:

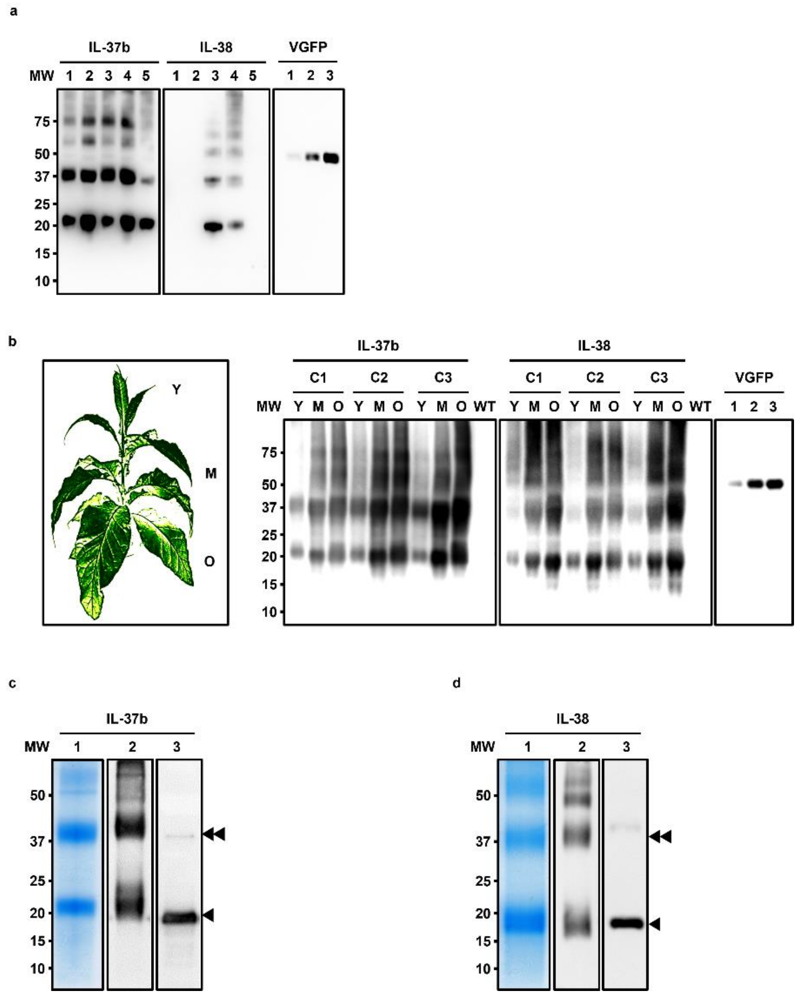

Original images (cropped parts are boxed in red).

Figure 1a.

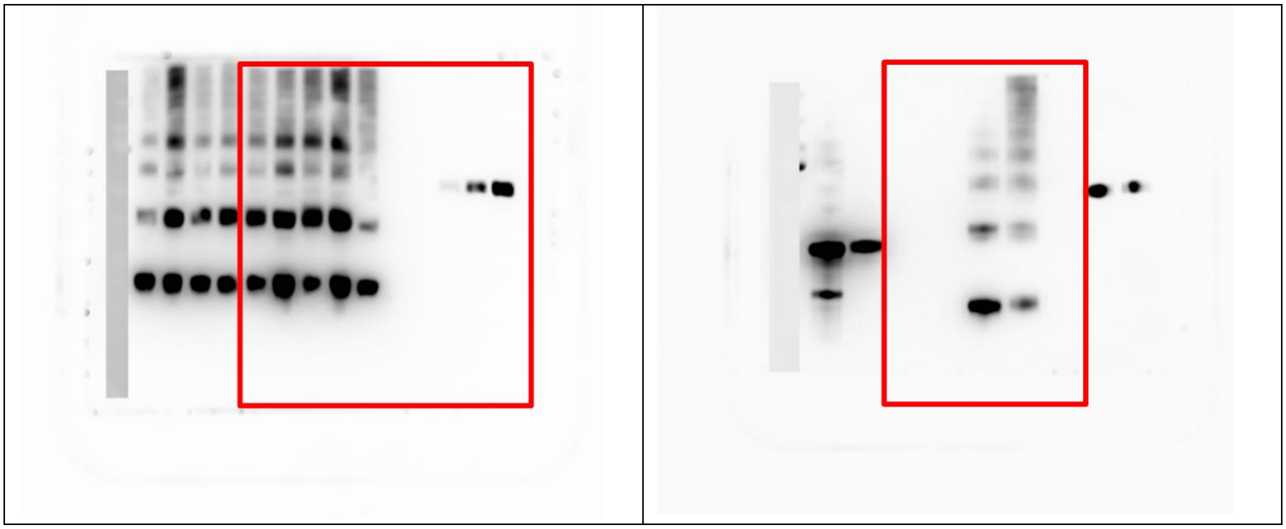

Figure 1b.

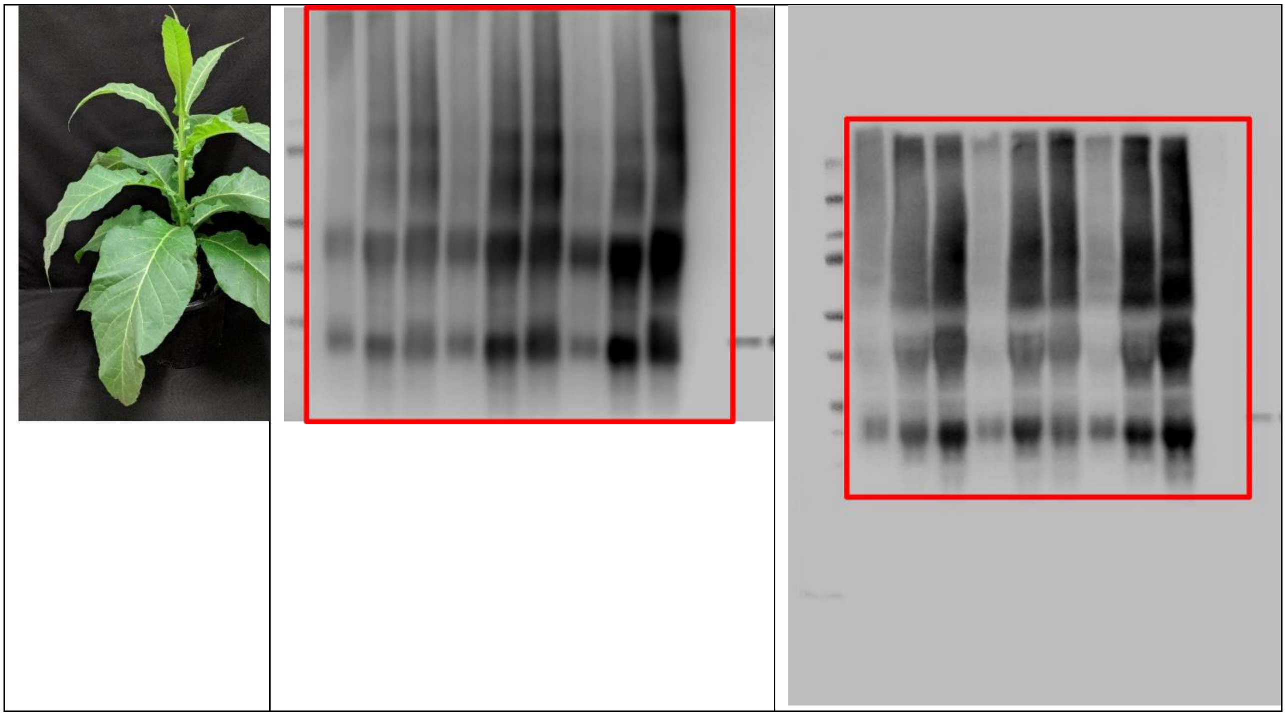

Figure 1c.

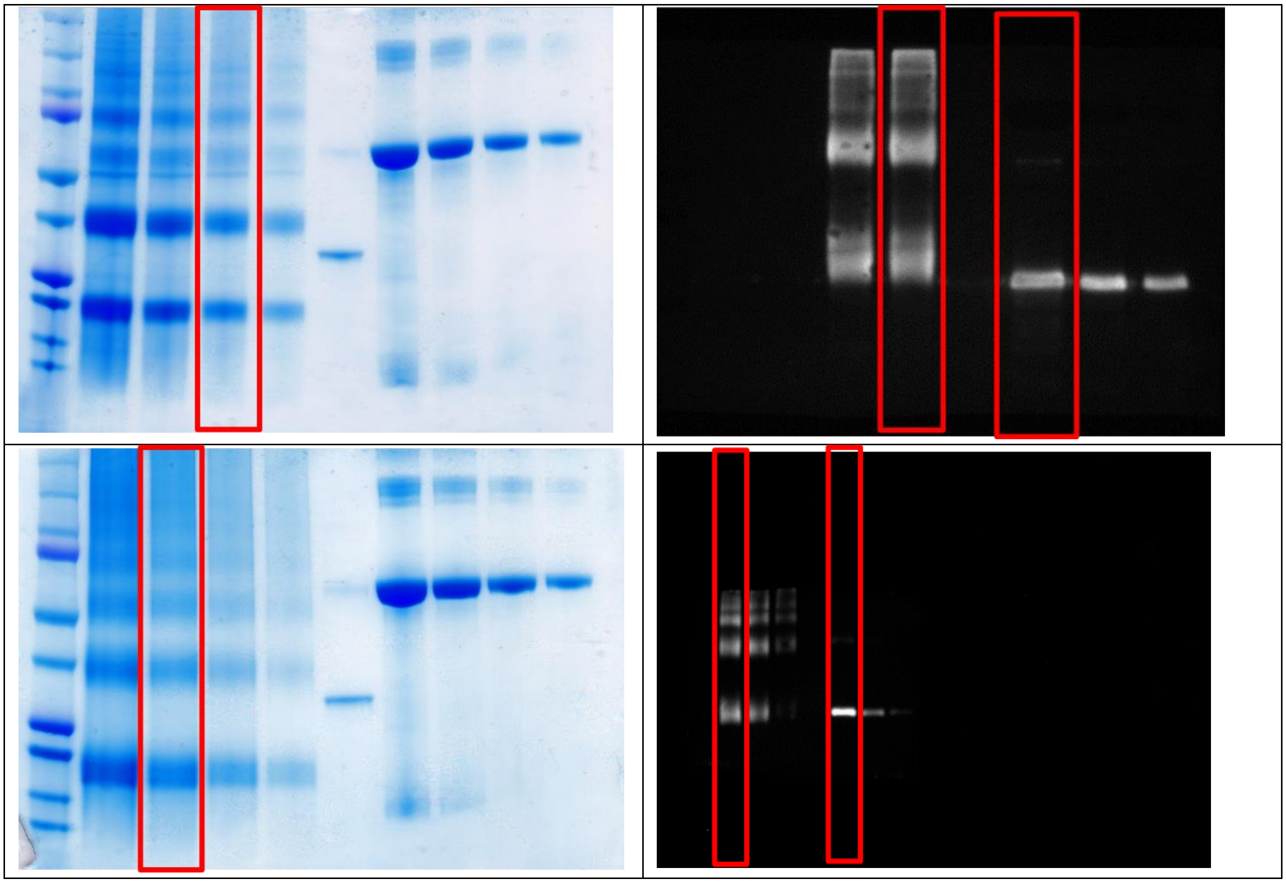

Supplement: Supplementary file 3 — Supplementary Information 3. [file 41598_2022_23828_MOESM3_ESM.pdf]
