# Supplementary material for: Plant-produced recombinant cytokines IL-37b and IL-38 modulate inflammatory response from stimulated human PBMCs
Source: Sci Rep. 2022 Nov 14;12:19450. doi: 10.1038/s41598-022-23828-z (PMC9663505; doi:10.1038/s41598-022-23828-z)
Supplement: Supplementary file 4 — Supplementary Information 4. [file 41598_2022_23828_MOESM4_ESM.pdf]

Supplementary Material – Table SI.

Manuscript: Plant-produced Recombinant Cytokines IL-37b and IL-38 Modulate Inflammatory Response from Stimulated Human PBMCs.

Igor Kolotilin,
Solar Grants Biotechnology Inc., London, Ontario, Canada.
igor.k@sgbiotec.com

Table SI. Results of GEE model analyses of modulation of inflammatory cytokines levels secreted from IA-stimulated PBMCs with applied treatments of plantakines IL-37b and IL-38.
The percentages represent the average effect of the plantakines treatments compared with the positive controls at corresponding concentrations. Calculated p-values are also displayed. IM – Inflammatory Marker.

**IM**	**Plantakines Treatments (ng/mL)**	**Inflammatory Agent (IA)**			
		**LPS, pg/mL**		**PHA, μg/mL**	
		**150**	**300**	**5**	**10**
GM-CSF	IL-37b (1)	-54.2%, p < 0.001	-25.3%, p < 0.001	19.6%, p = 0.013	-5.0%, p = 0.411
	IL-37b (10)	-11.3%, p = 0.024	59.2%, p < 0.001	50.5%, p < 0.001	-5.8%, p = 0.011
	IL-37b (100)	53.9%, p < 0.001	100.5%, p < 0.001	155.9%, p < 0.001	127.8%, p < 0.001
	IL-38 (1)	-59.4%, p < 0.001	-14.1%, p = 0.118	18.0%, p = 0.403	1.5%, p = 0.881
	IL-38 (10)	7.3%, p = 0.230	29.1%, p < 0.001	103.3%, p < 0.001	42.6%, p = 0.016
	IL-38 (100)	21.2%, p = 0.013	88.8%, p < 0.001	380.5%, p < 0.001	326.6%, p < 0.001
	IL-37b (10) × IL-38 (10)	78.7%, p < 0.001		228.6%, p < 0.001	
IFNγ	IL-37b (1)	-53.2%, p < 0.001	-39.3%, p < 0.001	-16.5%, p = 0.038	-7.7%, p = 0.125
	IL-37b (10)	-47.3%, p < 0.001	0.0%, p = 1.000	-39.3%, p < 0.001	-44.0%, p < 0.001
	IL-37b (100)	-21.3%, p = 0.009	-13.1%, p = 0.487	-40.5%, p < 0.001	-22.9%, p < 0.001
	IL-38 (1)	-63.9%, p < 0.001	-16.5%, p = 0.006	-35.6%, p = 0.137	-36.9%, p = 0.164
	IL-38 (10)	-16.5%, p = 0.012	-24.4%, p = 0.018	-53.2%, p = 0.032	-53.2%, p = 0.046
	IL-38 (100)	-40.5%, p = 0.008	-21.3%, p < 0.001	6.2%, p = 0.856	6.2%, p = 0.852
	IL-37b (10) × IL-8 (10)	4.1%, p = 0.907		4.1%, p = 0.883	
IL-1α	IL-37b (1)	-24.4%, p < 0.001	-11.3%, p = 0.006	-2.0%, p = 0.648	-2.0%, p = 0.701
	IL-37b (10)	-14.8%, p < 0.001	10.5%, p = 0.077	-7.7%, p = 0.125	-11.3%, p = 0.246
	IL-37b (100)	-7.7%, p = 0.068	8.3%, p = 0.298	10.5%, p = 0.327	19.7%, p = 0.012
	IL-38 (1)	-25.9%, p < 0.001	-7.7%, p = 0.178	-9.5%, p = 0.480	-5.8%, p = 0.469
	IL-38 (10)	-14.8%, p < 0.001	2.0%, p = 0.550	4.1%, p = 0.690	-2.0%, p = 0.893
	IL-38 (100)	-3.9%, p = 0.380	6.2%, p = 0.188	46.2%, p = 0.015	40.5%, p < 0.001

	IL-37b (10) × IL-38 (10)	15.0%, p = 0.110		35.0%, p = 0.024	
IL-1β	IL-37b (1)	-24.4%, p < 0.001	-16.5%, p < 0.001	-5.8%, p = 0.409	-2.0%, p = 0.550
	IL-37b (10)	-19.7%, p < 0.001	6.2%, p = 0.094	-13.1%, p = 0.021	-9.5%, p = 0.181
	IL-37b (100)	-16.5%, p = 0.127	-2.0%, p = 0.701	2.0%, p = 0.761	2.0%, p = 0.701
	IL-38 (1)	-27.4%, p < 0.001	-13.1%, p < 0.001	-2.0%, p = 0.888	0.0%, p = 1.000
	IL-38 (10)	-13.1%, p = 0.002	-2.0%, p = 0.550	4.1%, p = 0.801	4.1%, p = 0.761
	IL-38 (100)	-13.1%, p = 0.002	2.0%, p = 0.648	43.3%, p = 0.019	46.2%, p = 0.001
	IL-37b (10) × IL-38 (10)	17.4%, p = 0.264		35.0%, p = 0.030	
IL-6	IL-37b (1)	-9.5%, p = 0.012	-3.9%, p = 0.380	-5.8%, p = 0.409	-2.0%, p = 0.826
	IL-37b (10)	-2.0%, p = 0.701	6.2%, p = 0.323	-5.8%, p = 0.515	-9.5%, p = 0.181
	IL-37b (100)	8.3%, p = 0.178	8.3%, p = 0.329	0.0%, p = 1.000	4.1%, p = 0.711
	IL-38 (1)	-7.7%, p = 0.178	-5.8%, p = 0.323	-5.8%, p = 0.798	-2.0%, p = 0.898
	IL-38 (10)	0.0%, p = 1.000	2.0%, p = 0.736	10.5%, p = 0.538	6.2%, p = 0.722
	IL-38 (100)	0.0%, p = 1.000	0.0%, p = 1.000	15.0%, p = 0.432	4.1%, p = 0.810
	IL-37b (10) × IL-38 (10)	8.3%, p = 0.608		6.2%, p = 0.697	
IL-8	IL-37b (1)	-26.7%, p = 0.100	24.6%, p = 0.367	-14.8%, p = 0.356	35.0%, p = 0.391
	IL-37b (10)	-28.5%, p = 0.082	-11.3%, p = 0.264	22.1%, p = 0.552	6.2%, p = 0.832
	IL-37b (100)	6.2%, p = 0.370	15.0%, p = 0.511	68.2%, p = 0.343	15.0%, p = 0.544
	IL-38 (1)	22.1%, p = 0.625	-28.5%, p = 0.032	-11.3%, p = 0.736	-5.8%, p = 0.823
	IL-38 (10)	8.3%, p = 0.639	-5.8%, p = 0.469	8.3%, p = 0.772	-3.9%, p = 0.886
	IL-38 (100)	-9.5%, p = 0.077	46.2%, p = 0.500	10.5%, p = 0.731	-2.0%, p = 0.943
	IL-37b (10) × IL-38 (10)	-11.3%, p = 0.655		-5.8%, p = 0.818	
IL-22	IL-37b (1)	-7.9%, p = 0.555	-18.2%, p = 0.004	-54.0%, p < 0.001	-17.3%, p = 0.026
	IL-37b (10)	-36.0%, p = 0.016	-26.1%, p < 0.001	-40.3%, p = 0.003	-26.4%, p < 0.001
	IL-37b (100)	-13.4%, p = 0.078	-33.5%, p < 0.001	-51.2%, p < 0.001	-12.4%, p = 0.131
	IL-38 (1)	-38.1%, p = 0.065	-40.0%, p < 0.001	8.1%, p = 0.631	86.8%, p < 0.001
	IL-38 (10)	-29.4%, p = 0.008	-45.0%, p < 0.001	55.8%, p < 0.001	61.7%, p = 0.061
	IL-38 (100)	-23.7%, p = 0.241	-31.8%, p = 0.003	15.3%, p = 0.347	50.0%, p = 0.020
	IL-37b (10) × IL-38 (10)	46.0%, p = 0.084		5.9%, p = 0.670	
IL-12	IL-37b (1)	-4.0%, p = 0.832	11.0%, p = 0.534	42.1%, p = 0.055	9.1%, p = 0.617
	IL-37b (10)	21.8%, p = 0.574	2.8%, p = 0.759	-2.6%, p = 0.825	-36.6%, p < 0.001
	IL-37b (100)	19.7%, p = 0.372	-4.8%, p = 0.483	7.5%, p = 0.613	-11.0%, p = 0.091
	IL-38 (1)	14.9%, p = 0.607	1.9%, p = 0.906	63.3%, p = 0.020	22.2%, p = 0.476
	IL-38 (10)	51.1%, p = 0.522	10.6%, p = 0.586	107.0%, p = 0.079	8.8%, p = 0.750
	IL-38 (100)	17.2%, p = 0.544	17.4%, p = 0.271	123.0%, p < 0.001	74.3%, p = 0.002
	IL-37b (10) × IL-38 (10)	98.5%, p = 0.023		134.5%, p < 0.001	
IL-17	IL-37b (1)	0.0%, p = 1.000	-16.5%, p < 0.001	-16.5%, p = 0.105	12.7%, p < 0.001
	IL-37b (10)	-36.9%, p = 0.002	-5.8%, p = 0.094	-9.5%, p = 0.286	19.7%, p < 0.001
	IL-37b (100)	-35.6%, p = 0.003	-16.5%, p = 0.038	-3.9%, p = 0.510	15.0%, p < 0.001
	IL-38 (1)	-21.3%, p = 0.004	-28.8%, p < 0.001	4.1%, p = 0.807	22.1%, p = 0.166
	IL-38 (10)	-16.5%, p = 0.012	-31.6%, p < 0.001	24.6%, p = 0.215	19.7%, p = 0.188
	IL-38 (100)	-24.4%, p = 0.025	-30.2%, p < 0.001	29.7%, p = 0.107	55.3%, p = 0.003
	IL-37b (10) × IL-38 (10)	12.7%, p = 0.409		43.3%, p = 0.021	

TNFα	IL-37b (1)	-18.1%, p = 0.018	-9.5%, p = 0.627	-56.0%, p = 0.170	-25.9%, p = 0.399
	IL-37b (10)	-28.8%, p = 0.020	17.4%, p = 0.431	-60.1%, p = 0.109	0.0%, p = 1.000
	IL-37b (100)	-28.8%, p = 0.020	0.0%, p = 1.000	-45.1%, p = 0.020	35.0%, p = 0.145
	IL-38 1	-36.9%, p = 0.035	-49.3%, p = 0.184	46.2%, p = 0.173	55.3%, p = 0.411
	IL-38 10	-44.0%, p = 0.116	-40.5%, p = 0.311	49.2%, p = 0.123	166.4%, p = 0.105
	IL-38 100	-34.3%, p = 0.232	-13.1%, p = 0.581	85.9%, p = 0.026	252.5%, p = 0.017
	IL-37b (10) × IL-38 (10)	19.7%, p = 0.445		61.6%, p = 0.002	
IL-10	IL-37b (1)	-9.5%, p = 0.114	-14.8%, p < 0.001	-5.8%, p = 0.094	4.1%, p = 0.510
	IL-37b (10)	-9.5%, p = 0.012	10.5%, p = 0.012	-5.8%, p = 0.370	6.2%, p = 0.442
	IL-37b (100)	-22.9%, p < 0.001	-24.4%, p < 0.001	-25.9%, p < 0.001	-5.8%, p = 0.370
	IL-38 (1)	-14.8%, p = 0.040	6.2%, p = 0.006	17.4%, p = 0.244	22.1%, p = 0.033
	IL-38 (10)	-3.9%, p = 0.264	8.3%, p = 0.178	15.0%, p = 0.224	32.3%, p < 0.001
	IL-38 (100)	-30.2%, p < 0.001	-28.8%, p < 0.001	15.0%, p = 0.275	22.1%, p = 0.033
	IL-37b (10) × IL-38 (10)	35.0%, p = 0.021		43.3%, p < 0.001	
